# Shedding light on walking in the dark: the effects of reduced lighting on the gait of older adults with a higher-level gait disorder and controls

**DOI:** 10.1186/1743-0003-2-27

**Published:** 2005-08-28

**Authors:** Anat Kesler, Gregory Leibovich, Talia Herman, Leor Gruendlinger, Nir Giladi, Jeffrey M Hausdorff

**Affiliations:** 1 Movement Disorders Unit, Department of Neurology, Tel-Aviv Sourasky Medical Center, Tel-Aviv, Israel; 2 Department of Ophthalmology, Tel-Aviv Sourasky Medical Center, Tel-Aviv, Israel; 3 Department of Physical Therapy, Sackler School of Medicine, Tel-Aviv University, Tel-Aviv, Israel; 4 Department of Neurology, Sackler School of Medicine, Tel-Aviv University, Tel-Aviv, Israel; 5 Division on Aging, Harvard Medical School, Boston, MA, USA

**Keywords:** gait, variability, vision, fall risk, aging, lighting, Higher-Level Gait Disorders

## Abstract

**Objective:**

To study the effects of reduced lighting on the gait of older adults with a high level gait disorder (HLGD) and to compare their response to that of healthy elderly controls.

**Methods:**

22 patients with a HLGD and 20 age-matched healthy controls were studied under usual lighting conditions (1000 lumens) and in near darkness (5 lumens). Gait speed and gait dynamics were measured under both conditions. Cognitive function, co-morbidities, depressive symptoms, and vision were also evaluated.

**Results:**

Under usual lighting conditions, patients walked more slowly, with reduced swing times, and increased stride-to-stride variability, compared to controls. When walking under near darkness conditions, both groups slowed their gait. All other measures of gait were not affected by lighting in the controls. In contrast, patients further reduced their swing times and increased their stride-to-stride variability, both stride time variability and swing time variability. The unique response of the patients was not explained by vision, mental status, co-morbidities, or the values of walking under usual lighting conditions.

**Conclusion:**

Walking with reduced lighting does not affect the gait of healthy elderly subjects, except for a reduction in speed. On the other hand, the gait of older adults with a HLGD becomes more variable and unsteady when they walk in near darkness, despite adapting a slow and cautious gait. Further work is needed to identify the causes of the maladaptive response among patients with a HLGD and the potential connection between this behavior and the increased fall risk observed in these patients.

## Introduction

Many older adults have an impaired gait that does not appear to be a result of any well defined disease [1]. In their review of patients attending a neurology clinic, Sudarsky et al. reported that the cause of the gait disturbance was unknown, even after neuro-imaging, in about 10–20 percent of older adults with a disturbed gait [2,3]. In a study of the "oldest old" (age range 87 to 97 years) in the Netherlands, Bloem et al. observed that about 20 percent of those studied had a normal gait, 69 percent had a gait disorder due to known disease, and about 11 percent of the subjects had an idiopathic "senile gait disorder", i.e., a gait disorder of unknown origin [4]. Of note, those subjects with a gait disorder of unknown origin had a higher risk of mortality during a five year follow up period, compared to the group of age-matched subjects who had a normal gait [5], suggesting that the origin of this gait disorder is not benign.

Nutt et al. coined the term "higher-level" gait disorders (HLGD) to refer to an altered gait that is not a result of lower extremity or peripheral dysfunction and cannot be attributed to well defined chronic disease [6,7]. One common example of a HLGD is the idiopathic "cautious" gait of the elderly or the "senile gait" disorder [6,7]. A "cautious" gait is typically marked by mild to moderate slowing, reduced stride length, and mild widening of the base of support [7]. Previous studies have shown that older adults with a cautious gait and HLGD also walk with increased stride variability and unsteadiness, have an excessive fear of falling that appears to be related to this increased stride variability, and have an increased risk of falls [8,9]. Further, the extrapyramidal, limbic systems, and the frontal lobe apparently play an important role, to different degrees, in what can be viewed as a multi-system neurodegenerative syndrome clearly different from "aging" [8]. Indeed, a three year prospective study found that gait and function deteriorated to a much greater extent among older adults with a HLGD, compared to controls, supporting the idea that this is a progressive neurodegenerative disorder [10]. However, the origin of the cautious gait in older adults with a HLGD remains largely unknown.

The gait changes observed in patients with a HLGD have features common to subjects who walk in the dark or with impaired vision, i.e., to others who might be walking cautiously. When vision is altered or lighting is reduced, subjects typically adapt a slower gait [11-13]. Variability of foot placement, at least during gait termination, may also be increased when lighting is not adequate [12]. These changes are reminiscent of the walking pattern of older adults with a HLGD and cautious gait. An elevated risk of falling has been associated with visual impairments, a problem that increases with age [14-17] and fall risk has also been associated with inadequate lighting, but the effects of vision and lighting have not been studied in older adults with a HLGD. To more fully characterize the gait of older adults with a HLGD and their reliance on visual input, we examined the effect of lighting changes on the gait of older adults with a HLGD and compared their response to that of healthy elderly controls. More specifically, we hypothesized that the response to near darkness may exacerbate gait instability and fall risk markers in these patients.

## Methods

### Participants

Twenty-two older adults between the ages of 70 and 90 years old who met previously established criteria for a HLGD [8,9] were included in the present study. Patients were recruited from among those who came to the Geriatric Outpatient Clinic or the Movement Disorders Unit at the Tel Aviv Sourasky Medical Center for evaluation of walking difficulties of unknown origin. All patients were mobile and walked independently at the time of assessment and all underwent a thorough general and neurological examination to ensure that subjects met the criteria of HLGD.

Patients were excluded if the cause of their gait disturbance could be readily established. Thus, patients with a history of clinically established stroke, Parkinson's disease, Alzheimer's disease, possible normal pressure hydrocephalus or other diagnosed neurodegenerative disorder, and patients with rest tremor or pronounced bradykinesia were excluded. Patients who were taking anti-parkinsonian or anti-spastic medications, or had orthostatic hypotension were also excluded. We also excluded patients with significant visual, peripheral, or vestibular disturbances, as well as patients with significant orthopedic disturbances. Patients with dementia according to the DSM IV criteria [18], history of psychiatric disease, or past use of dopamine receptor blocking agents (anti-psychotic medications) were excluded as well. In addition, we excluded patients with a history of traumatic head injury and/or loss of consciousness. In brief, no specific disorder could be diagnosed as the cause of the patients' complaint about his or her walking difficulties.

The patient population was compared to a group of twenty healthy controls of similar age. Control subjects were recruited from the community and from nearby elderly housing facilities or were spouses of outpatients. Subjects were included if they were between 70 and 90 years of age, reported normal walking function, had no obvious clinical impairment, and did not have significant cognitive impairment (Mini Mental State Examination >25 [19]). Subjects were excluded if they had any neurological disorder or any significant clinical history likely to affect their gait (e.g., stroke).

The study was approved by the Human Studies committee of the Tel-Aviv Sourasky Medical Center. All subjects provided informed written consent according to the declaration of Helsinki prior to entering the study.

### Subject Characteristics and Assessment of Vision

The Mini Mental State Examination (MMSE) [19] and the Geriatric Depression Scale (GDS) [20] were administered to probe the mental health of the subjects. Body-mass-index (BMI) was determined and Charlson's co-morbidity index was used to quantify general health status; scores closer to zero reflect better health [21].

Three aspects of vision were evaluated: 1) visual acuity, using the Snellen vision chart, 2) color blindness, using Ishihara pseudochromatic color test [22], and 3) contrast sensitivity. Previous studies have indicated that visual acuity and contrast sensitivity, a robust indicator of functional vision [23], are associated with an increased risk of falls among the elderly [16,24-26]. Visual acuity scores were stratified in normal (i.e., good or mild decline, 6/6–6/12) and abnormal (>6/15). Contrast sensitivity was measured using a wall mounted clinical chart, a standard clinical tool (Vistech VCTS 6000). The chart contains 5 rows of 9 printed circular patches each of which displays a sine wave grating. There are 5 spatial frequencies across the 5 rows (1.5, 3, 6, 12, and 18 cycles per degree). The chart luminance was standardized according to the light meter supplied with the chart. The last patch on which the patient correctly identified the direction of the gratings was recorded for each frequency. For all tests, each eye was examined separately. The function of the better eye was used in all analysis, since both eyes were used during walking. The eye examinations were performed by a neuro-ophthalmologist who was blinded to the gait measures in a subset of subjects who were selected at random.

### Walking Protocol

Subjects were instructed to walk on level ground under usual lighting conditions (1000 lumens) and in near darkness (5 lumens). Although there are advantages to performing these tests in a random order, the usual lighting condition was always performed first. If anything, this should maximize safety and minimize the effects of the walking in darkness; since this condition is always performed second, subjects have more time to adapt to the environment and walking conditions. Between the two walks, subjects sat and rested for at least two minutes. In order to control the lighting conditions, testing took place in a large, quiet, empty room. The straight walking path was 9 meters long. Subjects walked along the path six times and were told to turn around and continue walking when they reached the end of the path. All subjects were tested in the same environment. Subjects were "guarded" by a research assistant who walked a few steps away from the subject, making sure not to interfere or set the pace. Study subjects were not aware of the specific questions of this investigation.

### Assessment of Gait Dynamics

Previously described methods were used to quantify gait variability and evaluate gait dynamics of each walk [9,27,28]. Briefly, to measure the gait rhythm and the timing of the gait cycle (i.e., the stride time and the swing time), a computerized force-sensitive system was used to evaluate gait and stride-to-stride variability [29,30]. The system measures the forces underneath the foot as a function of time. The system consists of a pair of shoes and a recording unit. Each shoe contains 8 load sensors that cover the surface of the sole and measure the vertical forces under the foot. The recording unit (19 × 14 × 4.5 cm; 1.5 kg) is carried on the waist. Plantar pressures under each foot are recorded at a rate of 100 Hz. Measurements are stored in a memory card during the walk and, after the walk, are transferred to a personal computer for further analysis. Subsequently, the digitized data were transferred to a computer workstation for analysis using software that extracts the initial and end contact time of each stride and determines stride and swing times. To focus on the assessment of the dynamics of continuous, "normal" walking and each subject's "intrinsic" dynamics and to ensure that the analysis was not influenced by atypical strides (e.g., the turning at the end of the room), a median filter was applied to each subject's time series to remove data points that were three standard deviations greater than or less than the median value [31]. Subsequently, the average stride time, average swing time, stride time variability, and swing time variability were determined. Variability was calculated using the coefficient of variation (CV) of each subject's stride time or swing time, e.g., (100 × standard deviation of stride time)/(mean stride time). Stride-to-stride variability reflects gait unsteadiness and arrhythmicity and has been shown to prospectively predict falls [31-34]. The time to walk the 54 meters, the walk time, was also measured. The values for the left and right feet were highly correlated; for brevity, we report values from only right foot.

### Statistical Analysis

Descriptive statistics are reported as mean ± SD or %. We used the Student's t and Chi-square tests to compare the patient and control subjects with respect to different background characteristics (e.g., age, gender, vision) and Spearman's correlation coefficient to quantify correlations among measures. To evaluate the effect of lighting on gait parameters and to compare the groups, we used Mixed Effects Models for repeated measures. For each gait parameter, a separate model was applied. The dependent variable was the gait parameter and the independent variables were the group (patients or controls), the walking condition (i.e., light or near darkness), and the interaction term group × lighting condition. A p-value ≤ 0.05 (two-sided) was considered statistically significant. All statistical analyses were performed using SPSS 11.5 and SAS 8.2 (Proc Mixed).

## Results

Table 1 summarizes the general characteristics of the two study groups. Patients and controls were similar with respect to age, gender, body-mass-index, and depressive symptoms. MMSE scores were slightly, but significantly lower in the patients, but all subjects from both groups scored a 26 or higher (i.e., they were non-demented). Charlson scores were higher in the patients, but the scores were generally low and close to 0 (low co-morbidity) in both groups. As shown in Table 2, measures of visual acuity, color blindness and contrast sensitivity were similar in the patients and the controls.

**Table 1:** Subjects characteristics*

	**Patients with HLGD (n = 22)**	**Controls (n = 20)**
Age (yrs)	80.7 ± 4.1	80.6 ± 6.3
Gender (% male)	73%	65%
Body-mass-index (kg/m^2^)	26.6 ± 4.9	25.1 ± 2.9
Mini Mental State Exam (MMSE)	28.1 ± 1.3	29.4 ± 0.9
Geriatric Depression Scale	5.6 ± 4.7	3.8 ± 2.6
Charlson Comorbidity Score	0.0 ± 0.0	0.5 ± 0.7

*Subject characteristics were not different in the two groups (p > 0.13), except that the MMSE and the Charlson score tended to be slightly different in the patients (p < 0.01). Values are mean ± SD or %, as indicated. HLGD: Higher-level gait disorder.

**Table 2:** Measures of vision in the two study groups*

	**Patients with HLGD (n = 11)**	**Controls (n = 15)**
Visual Acuity (% normal)	82%	73%
Color Vision Test	8.2 ± 2.5	8.5 ± 1.3
Contrast Sensitivity Test: low spatial frequency	4.8 ± 0.7	4.5 ± 0.7
Contrast Sensitivity Test: intermediate spatial frequency	4.4 ± 0.7	4.2 ± 1.2
Contrast Sensitivity Test: high spatial frequency	1.8 ± 1.5	1.7 ± 1.5

*p > 0.19 for all comparisons.

Under normal lighting conditions, HLGD patients took more time to complete the walk and walked with an increased stride time, reduced swing time, and increased stride-to-stride variability of the stride and swing time, compared to the control subjects (p < 0.01) (see Table 3). Compared to normal lighting conditions, both patients and controls required significantly more time to complete the walk when walking in near darkness (p < 0.005). Walk times increased by 14.3% in the controls and by 15.8% in the patients, in other words, by similar amounts (p = 0.828). Among the control subjects, walking in near darkness did not significantly affect the average stride time, the average swing time, or the stride-to-stride variability of these measures (p > 0.29).

**Table 3:** Effects of lighting on gait

	**Patients with HLGD (n = 22)**		**Controls (n = 20)**	
	Normal Lighting	Near Dark (P-value*)	Normal Lighting	Near Dark (P-value*)

Average Stride Time (sec)	1.30 ± 0.17	1.29 ± 0.15 (0.376)	1.17 ± 0.12	1.17 ± 0.12 (0.912)
Stride Time Variability (%)	5.6 ± 2.3	6.8 ± 2.3 (0.005)	3.6 ± 1.2	4.1 ± 1.9 (0.295)
Average Swing Time (%)	33.9 ± 2.7	32.5 ± 3.7 (<0.001)	35.7 ± 3.0	35.5 ± 3.2 (0.015)
Swing Time Variability (%)	7.0 ± 2.9	10.1 ± 4.7 (<0.001)	4.5 ± 2.4	5.1 ± 2.5 (0.365)
Walk Time (sec)	106.3 ± 44.2	124.4 ± 57.1 (<0.001)	72.8 ± 20.8	84.2 ± 33.5 (0.013)

*P-values shown in parentheses are based on within group comparisons between near dark and normal lighting. All measures of gait were different (p < 0.01) in the two subject groups, both under normal lighting and in near darkness. Walk time is the time to complete the 54 meter walk.

**Figure 1:**
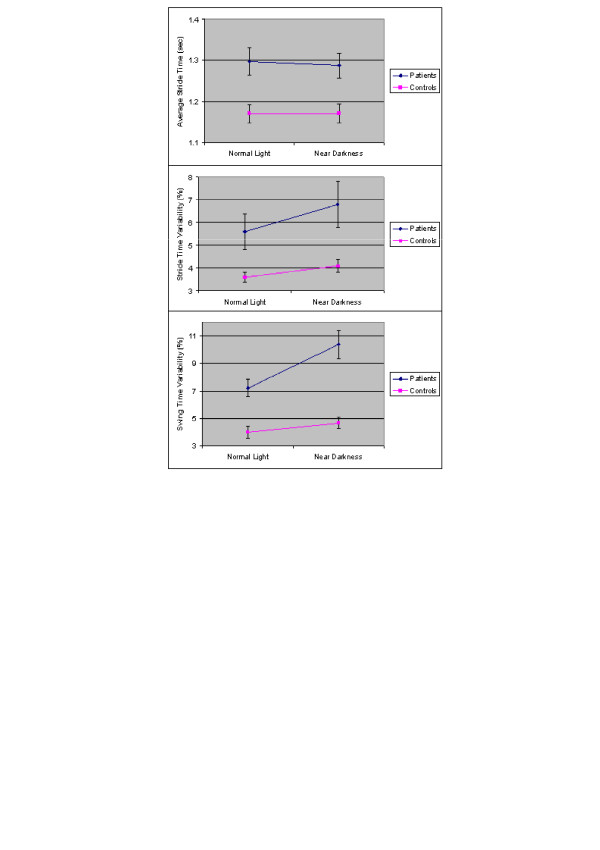
Effects of near darkness on stride time, stride time variability, and swing time variability in the two groups. For both groups, the average stride time was not affected by the change in lighting (p > 0.37). During walking in near darkness, variability measures were not significantly changed in the healthy controls (p > 0.29), but in the patients, stride time variability (p = 0.005) and swing time variability (p < 0.001) became significantly larger, compared to the values measured normal lighting.

In contrast to the control group, the gait of the patients with a HLGD became more abnormal when they walked in near darkness. There was no change in the average stride time when the patients walked in near darkness (p = 0.376), but the average swing time significantly decreased (p < 0.001), stride time variability increased (p = 0.005) and swing time variability increased (p < 0.001). As might be expected from Table 2, the group × lighting condition interaction term was significant for the average swing time (p = 0.015) and swing time variability (p = 0.005), indicating that near darkness affected the patients more than the controls (see Figure 1), despite similar increases in walk time. As noted above, all of the group differences in gait observed during normal lighting conditions persisted during walking in near darkness and the gap between the two groups widened.

Among the background and vision measures evaluated (e.g., age, gender, visual acuity, contrast sensitivity), the MMSE and the Charlson scores were the only measures that were significantly different and could, therefore, potentially mediate the group-specific changes in gait during walking in near dark. The MMSE was not correlated with the change in any of the gait measures observed during near dark walking (p > 0.17). Similarly, the Charlson scores did not explain the change in any of the gait measures (p > 0.07).

Subjects who took longer to complete the walk with normal lighting generally showed a relative increase in the walk time during near dark walking (r = 0.48; p = 0.001), whereas the changes in stride time variability and swing time variability were not significantly associated with the baseline values of these measures (p > 0.17). The change in stride time variability and swing time variability were moderately correlated with each other (r = 0.38; p = 0.018). The change in swing time variability was moderately correlated with the change in walk time (r = -0.40; p = 0.016), but the change in stride time variability was not correlated with the change in walk time (p = 0.12).

## Discussion

To summarize, key findings of this study are: 1) Both healthy older adults and older adults with a HLGD walk more slowly under diminished lighting conditions; 2) Diminished lighting does not increase the gait variability of healthy older adults; and 3) In patients with a HLGD, diminished lighting significantly increases gait variability. In the following paragraphs, we attempt to interpret these findings and discuss their implications for understanding HLGD, the role of vision in gait, and the relationship between visual impairment and increased fall risk in older adults.

Perhaps the simplest way to interpret the slower walking that occurs during diminished lighting conditions is that subjects are adapting a more cautious gait in response to the reduced lighting. This would be consistent with previous studies that suggest that gait slows down in the absence of sufficient visual input [11-13], perhaps to increase safety. This response of the healthy older adults to walking in near darkness also parallels the effects of an attention demanding task on the gait of healthy young and older adults [35,36]. When healthy young or older adults are asked to walk and perform an additional task simultaneously, gait speed is reduced, but there is no effect on gait variability [35,36]. From this perspective, one could suggest that walking in near darkness requires greater attention than walking under normal lighting.

When older adults with neurodegenerative disease or those with an increased risk of falls walk while simultaneously performing an attention demanding task, two things happen: 1) they slow down, like their healthy peers, and 2) stride variability increases [28,35-37]. These are the effects that were seen in the present study when the patients with a HLGD walked in near darkness. As noted, a possible explanation for this behavior, therefore, is that for the patients with a HLGD, walking in the dark is an attention demanding task. Alternatively, one could suggest that walking in near darkness reduces self-efficacy of walking in patients with HLGD, because they are already predisposed to fear of falling, but not in the controls, who do not have a marked concern about their gait. This could explain the disparate response of the two groups. Indeed, in older adults with a HLGD, fear of falling has been associated with stride time variability [9]. However, if this were the only contributing factor, one might have expected to see a larger reduction in walk times in the patient group, compared to the control, whereas the relative increases in walk times during near darkness were similar in the two groups.

Another potential explanation for the increased stride variability observed in the patients with a HLGD is based on the relationship between gait speed, stride length and stride frequency, on the one hand, and stride variability on the other [38-40]. At least in certain populations, some investigators suggest that variability of stride time and stride length becomes greater at slower walking speeds. One could argue that the increased variability observed in the patients with a HLGD in near darkness is simply a byproduct of their reduced walking speed. This explanation is, however, likely to be incomplete or incorrect. First, as noted, the healthy controls slowed down when walking in near darkness by virtually the same extent as that seen in the patient group, yet variability measures were unchanged in the controls. A reduced gait speed by itself does not necessarily lead to increased variability (as is the case for the response of healthy subjects to an attention demanding task, as discussed above). Second, in a study of healthy older adults and patients with Parkinson's disease, swing time variability was not affected by gait speed, even when gait speed was reduced by as much as 20% [30]. The increase in swing time variability observed among the patients with a HLGD during walking in near darkness cannot, therefore, be attributed to changes in gait speed.

Another way to view walking in near darkness is to consider it not simply as a test of vision, but as a challenge to other sensory feedback mechanisms that help to regulate gait. Walking may normally rely on visual, vestibular and proprioceptive feedback. When older adults or persons with deficits in balance are asked to close their eyes while standing on a balance platform, measures of sway and postural instability increase, both compared to eyes open conditions and compared to healthy young adults [41, 42]. These findings indicate that with aging, there is a greater reliance on visual feedback for maintenance of static balance; hence, when vision is removed, there is a large decrement in postural stability. We can apply similar reasoning to the present findings. In that case, one can interpret the results to suggest that in the patients with a HLGD, regulation of stride-to-stride variability relies on visual input and other feedback mechanisms are unable to fill in the gap that occurs when vision input is limited in near dark walking. This would suggest that patients with HLGD may have deficits in proprioception or vestibular function. Such deficits have not been identified in the present or previous studies of patients with HLGD [8,9]. It is possible, however, that these changes are relatively subtle and only surface when challenged.

The present study has several limitations. For example, we did not directly examine the affect of lighting on stress or fear of falling. Previous studies demonstrated that older patients with a HLGD have deficits in frontal lobe function, impairment in tests of balance and gait, and an increased risk of falls [8,9]. Future studies should assess if and how these factors contributed to the observed effects and how walking in darkness affects stress, anxiety and confidence in walking. It would also be helpful to evaluate other aspects of vision (e.g., peripheral vision) on a larger sample. We were not able to identify the specific factor that explained the increased sensitivity of the gait of patients with a HLGD to reduced lighting. Thus, the precise explanation for the further increase in stride-to-stride variability in near darkness in the patients with a HLGD remains to be determined.

Despite these limitations, the present findings shed light on the link between visual impairment, gait disturbances, and falls. Among older adults, falls are a major cause of morbidity and mortality [14]. Over one third of the adults aged 65 and over fall at least once each year [14] and among patients with a HLGD, falls are apparently much more frequent [9]. Previous studies have demonstrated that impaired vision is an important and independent risk factor for falls [14-17,24-26]. The present findings suggest a potential mechanism. With reduced vision or when walking in near darkness, perhaps two sides of the same coin, patients with an already increased risk of falls may further predispose themselves to falls and instability by increasing their stride-to-stride variability. A small perturbation could then take an already unstable system and cause a fall. Regardless of the precise explanation, the present results highlight the inappropriate response of patients with HLGD to reduced lighting conditions and suggest how this situation may aggravate gait instability and lead to falls in these older adults

## Conflict of interest statement

The author(s) declare that they have no competing interests.

## Contributors

A Kesler, G Leibovich, N Giladi, and JM Hausdorff designed the study. G Leibovich and T Herman participated in data collection. JM Hausdorff and L Gruendlinger helped with data analysis. A Kesler and JM Hausdorff drafted the manuscript. All authors helped with the interpretation of the results, reviewed the manuscript and participated in the editing of the final version of the manuscript.
